# Case Report: Durable Clinical Response to Third-Line Pyrotinib After Resistance to Trastuzumab in a Gastric Cancer Patient

**DOI:** 10.3389/fonc.2021.780577

**Published:** 2022-01-27

**Authors:** Junyi Wu, Lei Li, Jun Qin, Zhengqing Yan, Shiqing Chen, Tao Jin, Junming Xu

**Affiliations:** ^1^ Department of General Surgery, Shanghai General Hospital, Shanghai Jiaotong University School of Medicine, Shanghai, China; ^2^ The Medical Department, 3D Medicines Inc., Shanghai, China

**Keywords:** gastric cancer, HER2, trastuzumab, resistance, pyrotinib

## Abstract

**Background:**

Trastuzumab plus chemotherapy remains the standard first-line treatment strategy for HER2-positive gastric cancer (GC). Trastuzumab resistance, on the other hand, remains a significant issue. There are a few effective anti-HER2 agents for patients who develop resistance to trastuzumab.

**Case Presentation:**

A 49-year-old female was diagnosed with stage IV GC with liver and lung metastasis in July 2017. She underwent gastrostomy, and the immunohistochemistry (IHC) result of postoperative tissue demonstrated HER2 (3+). She received first-line treatment of trastuzumab (440 mg), oxaliplatin (200 mg), and S-1 (40 mg). After treatment for 6 months, the patient achieved complete response (CR) with PFS up to 21 months. After progression, she subsequently received trastuzumab (440 mg) plus oxaliplatin (200 mg) as second-line treatment. However, the patient developed resistance to trastuzumab after 12 months of treatment. She started to receive third-line treatment of irinotecan (200 mg d1) and capecitabine (60 mg bid) plus pyrotinib (400 mg/day). After 2 months of treatment, the tumor is evaluated as partial response with PFS of 12 months.

**Conclusions:**

We presented a patient with HER2-positive GC who benefited from the pyrotinib-based treatment after two lines of trastuzumab-based therapies failed. Further research is required to validate such conclusions.

## Introduction

Gastric cancer (GC) ranks the third among the leading causes of cancer deaths worldwide (1, 2). The survival outcomes in unresectable or advanced GC patients are poor with generally a 5-year survival rate of less than 20%. Although many clinical trials for GC treatment have been investigating the novel treatment strategies, most of them failed. Among various molecular biomarkers, human epidermal growth factor receptor 2 (HER2) remains a critical biomarker and accounted for ~5% to 36% GC (3). Although conflicting, some studies reported that HER2-positive related to the aggressive disease and poor outcomes.

Trastuzumab in combination with chemotherapy remains the standard first-line treatment strategy for HER2-positive GC. However, trastuzumab cardiotoxicity and resistance are two tricky issues (4, 5). Previous studies show that compensatory signal transduction of other HER receptors belong to a critical drug-resistance mechanism of trastuzumab (6). Currently, there are few effective anti-HER2 agents for patients who develop resistance to trastuzumab.

Pyrotinib, as a novel irreversible EGFR/HER2 dual tyrosine kinase inhibitor (TKI), has been approved by the National Medical Products Administration (NMPA) for HER2−positive breast cancer (7–9). A phase III randomized controlled trial demonstrated that pyrotinib plus capecitabine, compared with lapatinib plus capecitabine, could significantly prolong progression-free survival (PFS) (12.5 vs. 6.8 months) in patients with HER2-positive advanced breast cancer (10). Notably, patients achieved benefits from pyrotinib therapy, no matter whether trastuzumab was administered previously. Nine patients with HER2-positive GC were enrolled to receive pyrotinib-based therapy with a median OS of 5.9 months (95% CI: 4.0–9.6 months) (11). Currently, it is still unclear whether pan-HER inhibitor pyrotinib is an effective agent for trastuzumab-resistant GC. Herein, we presented a HER2-postive advanced GC that achieved durable clinical response to third-line pyrotinib, after two lines of trastuzumab-based treatments failed.

## Case Presentation

**Figure 1:**
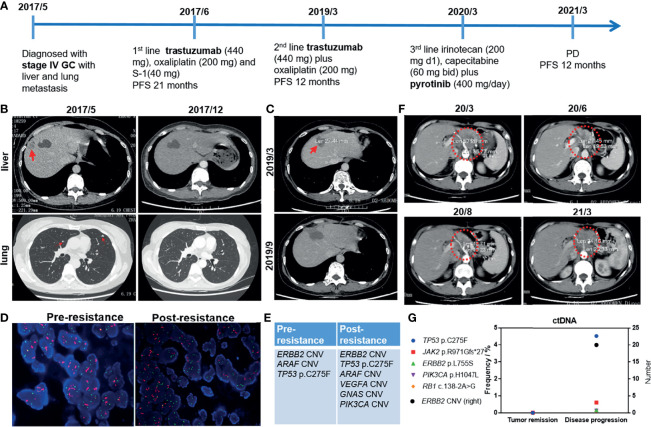
**(A)** The treatment procedure of the 49-year-old female with stage IV GC. GC, gastric cancer; PFS, progression-free survival; PD, progressive disease. **(B)** The results of chest computed tomography (CT) scans suggested that liver and lung lesions were reduced in size after first-line treatment. **(C)** CT results at first relapse in March 2019 and after surgical operation (R0 resection) in September 2019. **(D)** Fluorescence *in situ* hybridization (FISH) images demonstrated HER2 amplification. **(E)** NGS analysis of tumor tissue at diagnosis (pre-resistance) and first relapse (post-resistance). **(F)** The CT results following second relapse suggested the tumor shrank with third-line treatment. March 2020, Baseline; June and August 2020, during tumor remission; March 2021, tumor progression. **(G)** The ctDNA status during tumor remission and disease progression.

A 49-year-old female was diagnosed with stage IV GC with liver and lung metastasis in July 2017 (**Figure 1A**). She suffered gastrostomy and the immunohistochemistry (IHC) result of postoperative tissue demonstrated HER2 (3+). She received first-line treatment of trastuzumab (440 mg), oxaliplatin (200 mg), and S-1 (40 mg). After 6 months of treatment, the patient achieved complete response (CR) with PFS up to 21 months (**Figure 1B**). In March 2019, enlarged hepatic portal lymph nodes were observed. Liver metastasis lesion was curatively resected (R0) and subsequently second-line treatment of trastuzumab (440 mg) plus oxaliplatin (200 mg) was administrated (**Figure 1C**). The PFS was 12 months with enlarged peritoneal lymph nodes in March 2020, which suggested that the patient developed resistance to trastuzumab. Regardless of pre-resistance or post-resistance, both the tumors were HER2-positive (**Figure 1D**). The NGS results of the primary and liver lesions are shown in **Figure 1E**, respectively. The primary lesion harbored *HER2* copy number variation (CNV), *ARAF* CNV, and *TP53* p.C275F. Besides, *VEGFA*, *GNAS*, and *PIK3CA* CNVs were also found in the liver metastasis. The adverse effect (AE) was bone marrow suppression (Grade 2) during the treatment of trastuzumab plus chemotherapy. After multi-disciplinary team (MDT) discussion, she started to receive third-line treatment of irinotecan (200 mg d1) and capecitabine (60 mg bid) plus pyrotinib (400 mg/day). After 2 months of treatment, the tumor is evaluated as partial response (PR, **Figure 1F**). No ctDNA alterations are detected at tumor remission (**Figure 1G**). The AEs were diarrhea (Grade 1) and bone marrow suppression (Grade 2) during the third-line treatment. Until March 2021, progressive disease was observed with PFS of 12 months and the maximum allele frequency (MAF) of ctDNA was up to 4.53%. The patient was administrated trastuzumab + anti-PD-1 + chemotherapy as fourth-line treatment. Until the last follow-up in November 2021, no progressive disease was observed. Currently, close follow-up is still ongoing.

## Discussion

In this work, a pyrotinib-based regime was administered as third-line treatment for a HER2-positive GC patient who developed resistance to trastuzumab, and this patient achieved partial response. During treatment, ctDNA was used for monitoring tumor development. Interestingly, no ctDNA was detected during tumor remission. In contrast, the maximum allele frequency (MAF) of ctDNA was up to 4.53% (*TP53* p. C275F) during progression. Such results supported that ctDNA might be an alternative clinical biomarker for disease monitoring in GC.

With the development of non-invasive ctDNA sequencing technology, real-time monitoring tumor load is becoming an effective complement for tissue testing (12–14). Such technology has been used to monitor minimal residual disease (MRD), as well as tumor recurrence in various cancers, such as NSCLC and CRC (15, 16). Additionally, ctDNA response could be used to evaluate therapeutic effect and to explore potential resistance mechanisms for targeted drugs (17, 18). Considering the high heterogeneity characteristic of GC, ctDNA was used as an important tool for monitoring disease progression (19, 20). The appearance of ctDNA could predict tumor recurrence earlier than routine imaging examination. Previous work reported that ctDNA appearance during longitudinal post-operative follow-up was associated with worse DFS (HR = 14.78) and OS (HR = 7.664) (21). Furthermore, the clearance of ctDNA was associated with better clinical outcomes in advanced solid cancers, especially for the patients who were treated with pembrolizumab (14). In this work, no ctDNA was detected during tumor remission, and the maximum allele frequency (MAF) was up to 4.53% (*TP53* p. C275F) during progression. The present work highlighted that monitoring ctDNA might be a viable alternative to tissue-based genotyping in the metastatic setting.

Currently, HER2-targeted regimes have been widely used in various tumor treatments. HER2 protein plays a critical role in the tumorigenesis, tumor progression, and tumor metastasis (22). The ToGA trial suggested that trastuzumab combined with chemotherapy could significantly improve survival outcomes for advanced HER2-positive GC (23). However, the heterogeneity of GC and trastuzumab resistance limited the therapeutic effect of trastuzumab in clinical practice. Currently, some novel anti-HER2 agents (e.g., lapatinib, afatinib, neratinib, dacomitinib, pertuzumab, and ado-trastuzumab emtansine) are being investigated in HER2-positive GC, especially for those patients who progressed on or after trastuzumab-based therapy (24). Besides HER2, pan-HER inhibitors could induce sustained inhibition of HER3 or EGFR, which might overcome intrinsic or acquired resistance of trastuzumab. Such differences might support durable clinical response to pyrotinib in trastuzumab-resistant GC. Previous work reported that HER2-positive GC who received trastuzumab-based therapy also could benefit from 8th-line treatment of pyrotinib, a novel irreversible pan-HER TKI inhibitor (25). In this case, pyrotinib was administrated after two lines of trastuzumab, which might explain the efficacy of pyrotinib in trastuzumab-resistant GC better.

Previous studies indicated that bypass activation represented an important resistance mechanism for trastuzumab resistance (6). The RAS or PI3K signaling pathway, as a downstream signaling pathway of the HER2 receptor, is associated with intrinsic and/or acquired trastuzumab resistance and poor survival outcomes in patients who received trastuzumab treatment. In this case, *VEGFA*, *GNAS*, and *PIK3CA* CNVs were observed in tissue during trastuzumab resistance. The new emerging gene alterations *PIK3CA* p.H1047L, *HER2* p.L755S, *JAK2* p.R971Gfs*27, and *RB1* c.138-2A>G were observed in ctDNA during pyrotinib progression. Such results indicated that the resistance mechanism of trastuzumab and pyrotinib might be different. Furthermore, exploring their exact resistance mechanism is important and necessary. In view of the nature of case reports, such results should be further explored in larger cohorts.

## Conclusion

In this case, a pyrotinib-based regime was used as third-line therapy for a HER2-positive GC patient who developed resistance to trastuzumab, and the patient achieved a partial response. Furthermore, longitudinal ctDNA sequencing could be used to investigate drug resistance mechanisms and guide the precision treatment for GC patients. Further research is required to validate such conclusions.

## Ethics Statement

Written informed consent was obtained from the patient for the publication of any potentially identifiable images or data included in this article.

## Author Contributions

JX and TJ contributed to the planning and organization. JW and LL collected clinical data and supervised the findings of this work. JQ aided in the data collection and the supervision. ZY and SC analyzed the results and prepared the manuscript. All authors contributed to the article and approved the submitted version.

## Conflict of Interest

Authors ZY and SC were employed by the company 3D Medicines Inc.

The remaining authors declare that the research was conducted in the absence of any commercial or financial relationships that could be construed as a potential conflict of interest.

## Publisher’s Note

All claims expressed in this article are solely those of the authors and do not necessarily represent those of their affiliated organizations, or those of the publisher, the editors and the reviewers. Any product that may be evaluated in this article, or claim that may be made by its manufacturer, is not guaranteed or endorsed by the publisher.
